# Evaluating a Strengths-Based mHealth Tool (MyStrengths): Explorative Feasibility Trial

**DOI:** 10.2196/30572

**Published:** 2021-11-17

**Authors:** Stian Jessen, Jelena Mirkovic, Elanor Halvorsen Brendmo, Lise Solberg Nes

**Affiliations:** 1 Department of Digital Health Research Division of Medicine Oslo University Hospital HF Oslo Norway; 2 Institute of Clinical Medicine Faculty of Medicine University of Oslo Oslo Norway; 3 Department of Psychiatry and Psychology Mayo Clinic College of Medicine and Science Rochester, MN United States

**Keywords:** mHealth, personal strengths, gameful design, gamification, user engagement, explorative, feasibility, usefulness, usability, design, self-management, chronic illness

## Abstract

**Background:**

As the number of people living with chronic illnesses increases, providing wide-reaching and easy-to-use support tools is becoming increasingly important. Supporting people in this group to recognize and use more of their personal strengths has the potential to improve their quality of life. With this in mind, we have developed the MyStrengths app prototype, a gamefully designed app aimed at aiding users in both identifying their strengths and using these strengths more actively in their daily life.

**Objective:**

The goal of this study was to evaluate the user-reported feasibility and usefulness of the MyStrengths app. The study additionally aimed to explore whether the use of MyStrengths could be associated with selected psychosocial outcomes.

**Methods:**

A 31-day explorative feasibility trial with a pretest-posttest design and an optional end of study interview was conducted. Data collection included system-use log data, demographic information, pre– and post–psychosocial measures (ie, strengths use, self-efficacy, health-related quality of life, depression), user experience measures (ie, usability, engagement, flow), and interview data.

**Results:**

In total, 34 people with at least 1 chronic condition were enrolled in the study, with 26 participants (mean age 48 years, range 29-62 years; 1 male) completing the trial. Among these individuals, 18 were also interviewed posttrial. Participants used the MyStrengths app an average of 6 days during the trial period, with 54% (14/26) using the app over a period of at least 19 days. In total, 8738 unique app actions were registered. Of the psychosocial outcome measures, only 1 subscale, general health in the RAND 36-Item Health Survey, yielded significant pre- and posttest changes. Posttrial interviews showed that the number of participants who considered the MyStrengths app to be useful, somewhat useful, or not useful was evenly distributed across 3 groups. However, every participant did voice support for the strengths approach. All participants were able to identify a multitude of personal strengths using the MyStrengths app. Most participants that reported it to be useful had little or no previous experience with the personal strengths approach. A multitude of users welcomed the gameful design choices, particularly the rolling die feature, suggesting strengths exercises, activities that use a specific strength, were well received.

**Conclusions:**

Although the reported usefulness and feedback from use varied, most participants were favorable to the strengths-focused approach to care and support. Consequently, low-threshold and wide-reaching mobile health tools that use a strengths-focused approach, such as MyStrengths, hold the potential to support people living with chronic illness in performing self-management and achieving mastery of their life.

## Introduction

### Background

The number of people living with 1 or more chronic illnesses is increasing [1,2]. A recent review reported that up to 58% of people in developed countries live with multiple chronic diagnoses [3]. As the number of people living with chronic illness increases, finding new and more efficient ways of supporting them in living the best life possible with their illness is imperative. Self-management, or the activities, tasks, and skills one undertakes to manage life with illness, is one important aspect of such approaches [4]. Programs aiming to support and improve self-management often involve training and support that may aid in coping well with life with illnesses and include monitoring symptoms, adhering to medication regimens, and learning coping strategies [4,5].

Adding to these types of self-management approaches, research over the past few decades has also pointed to the potentially complementary benefits of exploring more “positive oriented“ approaches to health care and nursing, such as focusing on people's personal strengths when aiming to encourage, foster, and support self-management in chronic illness [6-9]*.*

### Personal Strengths

The concept of personal strengths has its foundation in positive psychology [7,10]. It has been defined as ”traits/capabilities that are personally fulfilling, do not diminish others, are ubiquitous and valued across cultures, and aligned with numerous positive outcomes for oneself and others“ [11]. Colloquially phrased, focusing on strengths means emphasizing what is valuable, possible, and doable, as opposed to the problem- and deficit-focused approach often present in modern medicine [12,13]. Being aware of and mobilizing one's strengths may lead to a wide range of positive effects for people in the general population, such as well-being and better quality of life [14]. More specifically, Seligman et al [7] found related increases in happiness and decreases in depression, Proyer et al [15] found increases in well-being and happiness, Linley et al [16] found better goal accomplishment, and Lee et al [17] found increased resilience in the face of challenges. Similarly, Wood and colleagues [18] connected strengths use in general to well-being, vitality, self-esteem, positive affect, and reductions in stress.

Although much of the strengths-related research has included participants from the general population [11,19], previous studies from our research group [12,20,21] have identified a multitude of strengths important to people living with chronic illnesses. In addition to items common to general strengths classifications, such as the Values In Action strengths classification [11] (eg, being kind and caring, persistent, having a positive outlook on life, or having courage), people with health challenges have also reported strengths vital to them to include support from family and peers, positive relationships with health care providers, and having helpful and constructive self-management strategies.

### Strengths Activities and Exercises

Strengths are not a static part of an individual's being but rather something malleable that can be changed and developed [11]. Nevertheless, although research related to activities that support people in recognizing and using their strengths, also called “strengths exercises*,*” have been published in the last few decades [11,22], few such studies and publications have focused on the potential of these strengths approaches for persons with chronic illnesses. Reviewing literature during the early phases of this project, our research team [23] identified 6 interventions described in 7 publications that included a personal strengths focus in chronic illness management and care [24-30]. The strengths activities used in these interventions included questionnaires asking participants to select strength(s) that apply the most to them [25,28,29], nominate their own strengths [26], use a specific strength in an activity each day [28,29] or to, in writing, reflect upon how they have used their strengths recently [24,26]. Although none of the studies reported on the development or adaption of the included strengths activities, the reported activities are still in line with common approaches for identifying and employing personal strengths [7,11,31-33]. These common approaches include using strengths in new and creative ways [7]; “strengths spotting*,*” which involves reflecting on how specific strengths are impacting you or trying to identify strengths in others [11,33]; the “three good things in life exercise” involving a daily routine of writing down three things that happened that day that you are grateful for and why [7,34]; and finally, performing “random acts of kindness” involving doing something that benefits others without directly benefiting oneself [35,36]. As with the outcomes of strengths use in general, as presented in the previous section, the identified studies employed a variety of outcome measures, including well-being [25-27,29,30], positive and negative affect [24,25,27], depression [24-26,29], strength-related outcomes such as self-esteem [27], and self-efficacy [30].

### Strengths Support for People With Chronic Illness

Although “people living with chronic illness” reflect a large and heterogenous group, many of the challenges faced in daily life, such as fatigue, sleeping problems, difficult emotions, or energy loss, are shared across diagnoses [37]. Aiding people living with chronic illnesses to be aware of and use their personal strengths more may lead to better wellness outcomes [38], and interventions aiming to help increase people's knowledge and use of their strengths may therefore be of great value for people living with chronic illnesses.

Both general or illness-specific patient educational activities and self-management training have been shown to benefit the patients with, for instance, improved self-management strategies and greater awareness of their condition [5], and to have positive health-economic impacts [39]. The majority of such programs appear to take place in municipalities and communities, while approximately one-third are hospital based [5]. These programs are often group based and peer led, and the content varies, and may include providing general and diagnose-specific information, learning from and sharing with others in similar situations, or conducting exercise sessions [40]. These programs are also available in Norway, where this study was conducted, and are often in the form of lifestyle courses that seek to improve participants' quality of life by aiding them in finding unrecognized resources within themselves and strengthening their copings skills [5].

Still, not everyone living with chronic illness has the opportunity to participate in self-management training, and some have also reported feelings of loss or the need for follow-up sessions after the programs have ended [5]. Owing to the ubiquity of smartphones and other mobile devices, mobile health (mHealth) tools hold potential for reaching a much larger audience than do in-person interventions and other activities with care providers [41]. Given this, mHealth can add channels to existing services and also reach underserved populations or otherwise not active users of existing learning or educational programs [41].

Over the past few decades, an ever-increasing number of mHealth tools have been developed for various target groups, including weight [42] and stress management [43], medication adherence [44], smoking cessation [45], and oral hygiene [46]. Whereas traditional in-person health interventions have the benefits of health care providers or support personnel providing individual guidance, mHealth tools, as the name implies, are typically available to the user anywhere and anytime. Availability does not guarantee use, however, and these tools also need to be designed in ways that users find helpful, motivating, and engaging [47-49]. A popular approach to increase users' engagement with apps in general—and for mHealth specifically—has for the past decade been to create more playful and gamelike user experiences using design approaches and techniques known from the world of games [50-52]. Creating such gameful designs typically includes using features such as competitions, collaborations, narratives, or immersive visual designs to increase the users' value creation and enjoyment [50,53,54].

In sum, finding ways to provide and deliver strengths-focused support to people with chronic illnesses through personal digital devices could benefit users and widen the overall service reach in potentially cost-efficient ways.

### The MyStrengths Project and App

Through earlier projects at our department, we have reported on multiple aspects related to the strengths approach to care and self-management including on the conceptualization of strengths and health assets [38], patients insights into and requirements of the strengths-approach [21], and the strengths reported by patients [12,20]. With a high degree of participation from people in the user group, our research group created the MyStrengths app. The development process and design activities (eg, idea-generating and design workshops with people in the user group, seminars with an international advisory group of researchers, and workshops with game and health technology designers) have been described in detail in previous publications [23,55-57].

**Figure 1:**
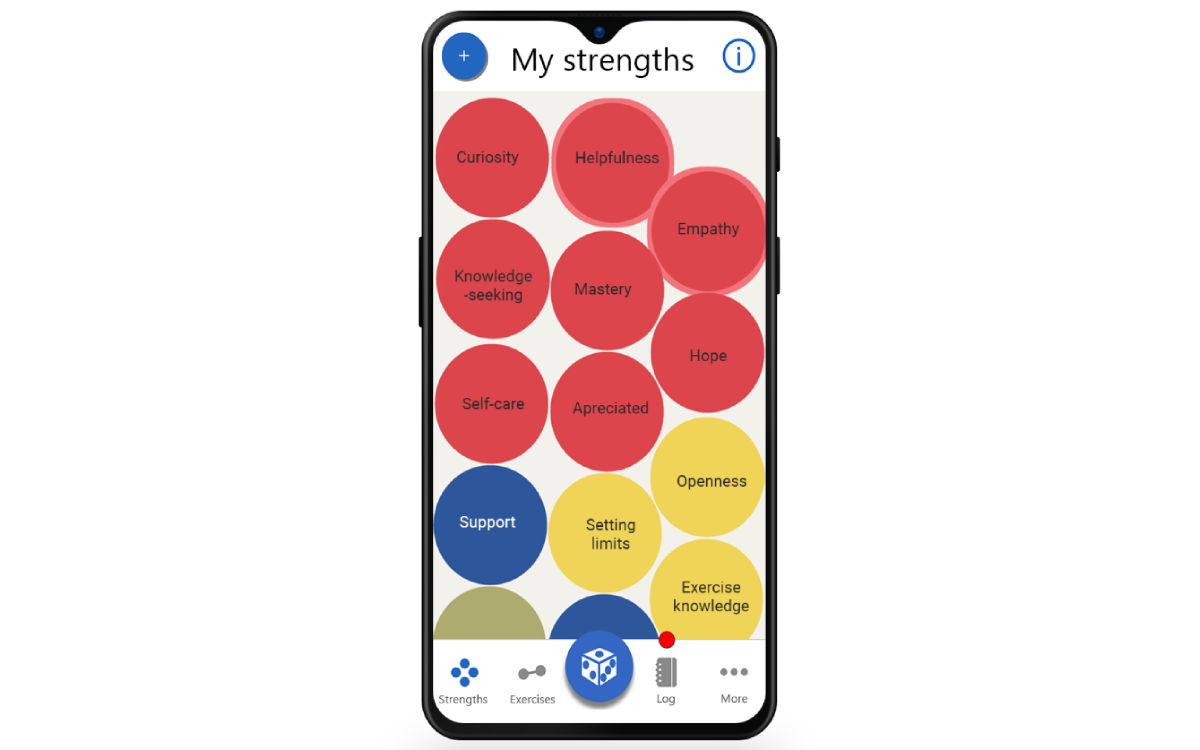
MyStrengths home screen.

**Figure 2:**
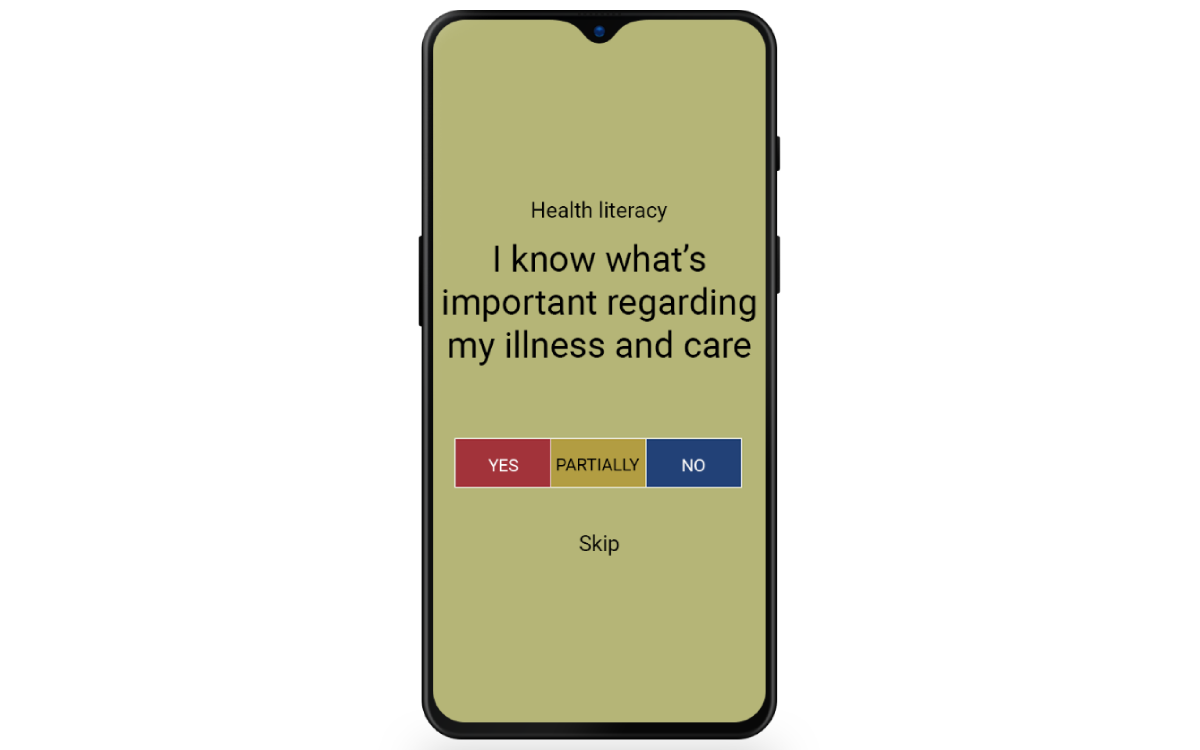
Strengths assesment.

**Figure 3:**
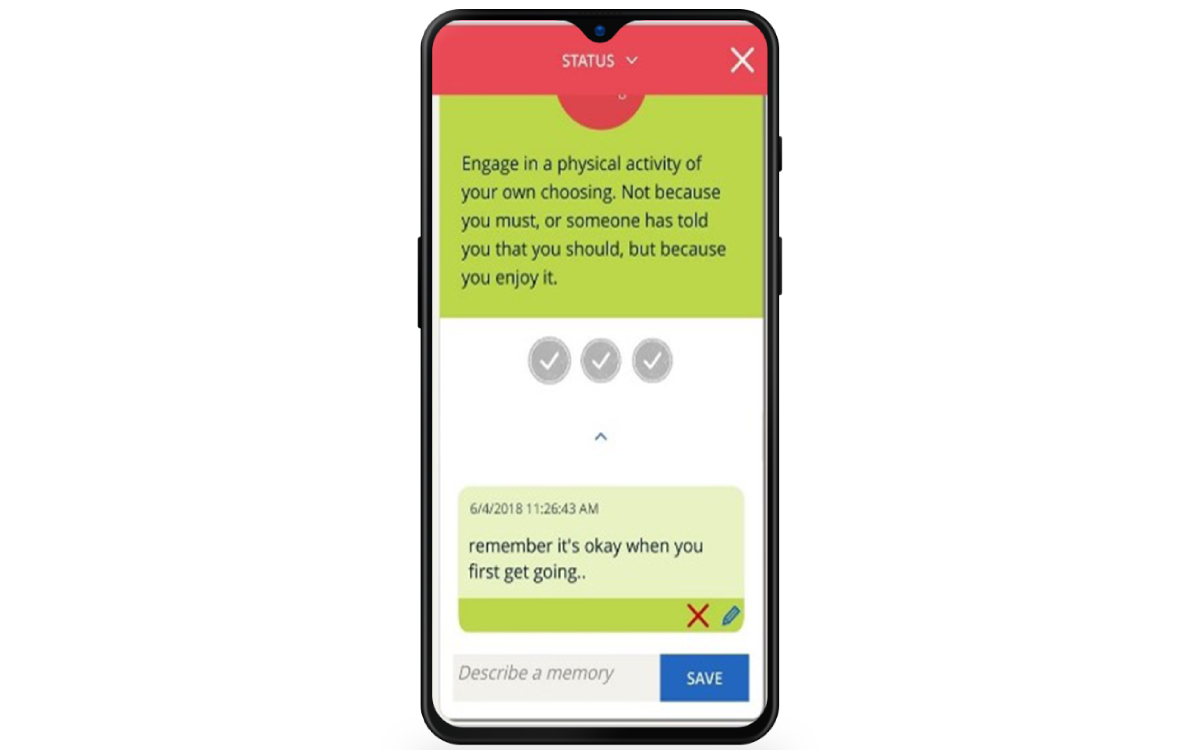
Exercise, with comment.

**Figure 4:**
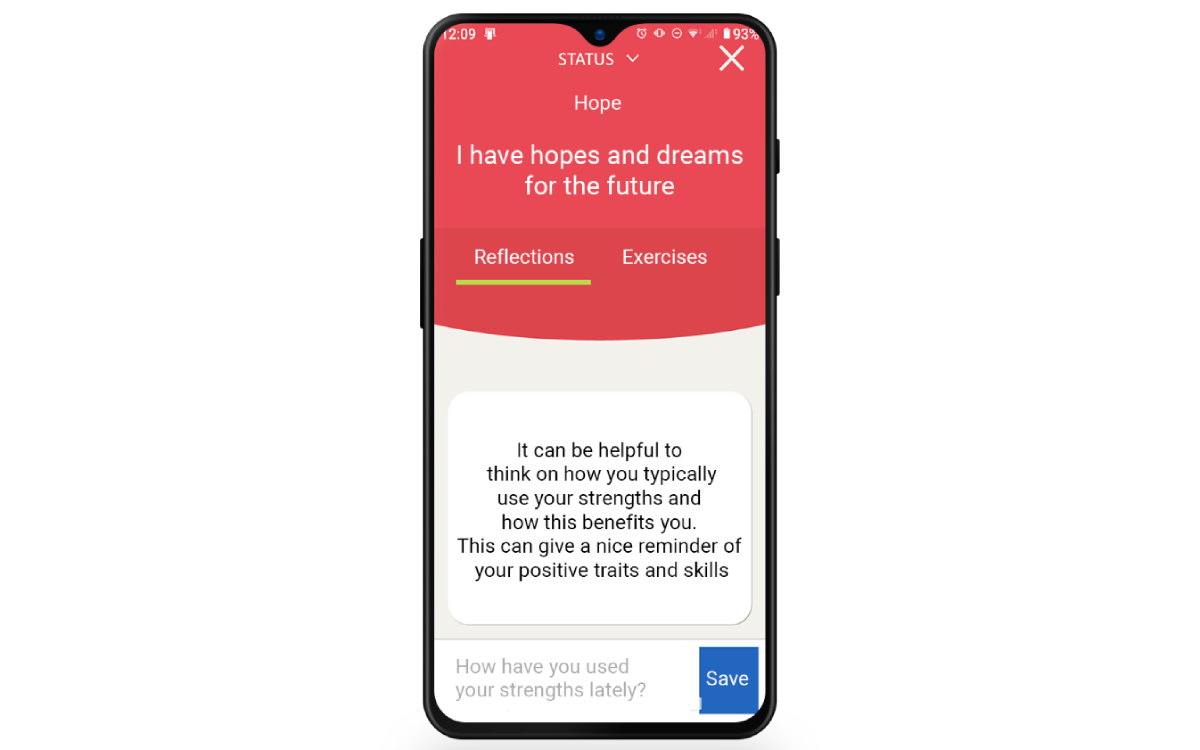
Reflections on strength.

The MyStrengths app is designed to help its users identify and use more of their strengths. Its main feature is an assessment and subsequent overview of the users' strengths. Each of the strengths preprogrammed into MyStrengths—40 in total—is presented as a sphere floating on the home screen (Figure 1). The details regarding the development and identification of the 40 strengths chosen can be found in the work by Kristjánsdóttir et al [12]. Unassessed strengths are shown as empty green spheres that float up on the screen, a few at a time. When a strengths item is clicked on and opened (as seen in Figure 2), it can be read in full and rated as either yes (having the strength), partially (partially having the strength), or no (not having the strengths); or the user can skip the strength. Depending on how the individual strengths are rated, they are colored red (having), yellow (partially), blue (not having), or green (skipped). Should the users feel that a strength is missing from the list, they can add additional strengths themselves. Having assessed ones' strengths, the app will suggest 2 to 3 strengths exercises for how to use and develop these (see Figure 3). When planned exercises are marked as completed, a short burst of celebratory stars showers the screen. The app also allows users to write a short reflection on the exercise and the strength used (see Figure 4).

**Figure 5:**
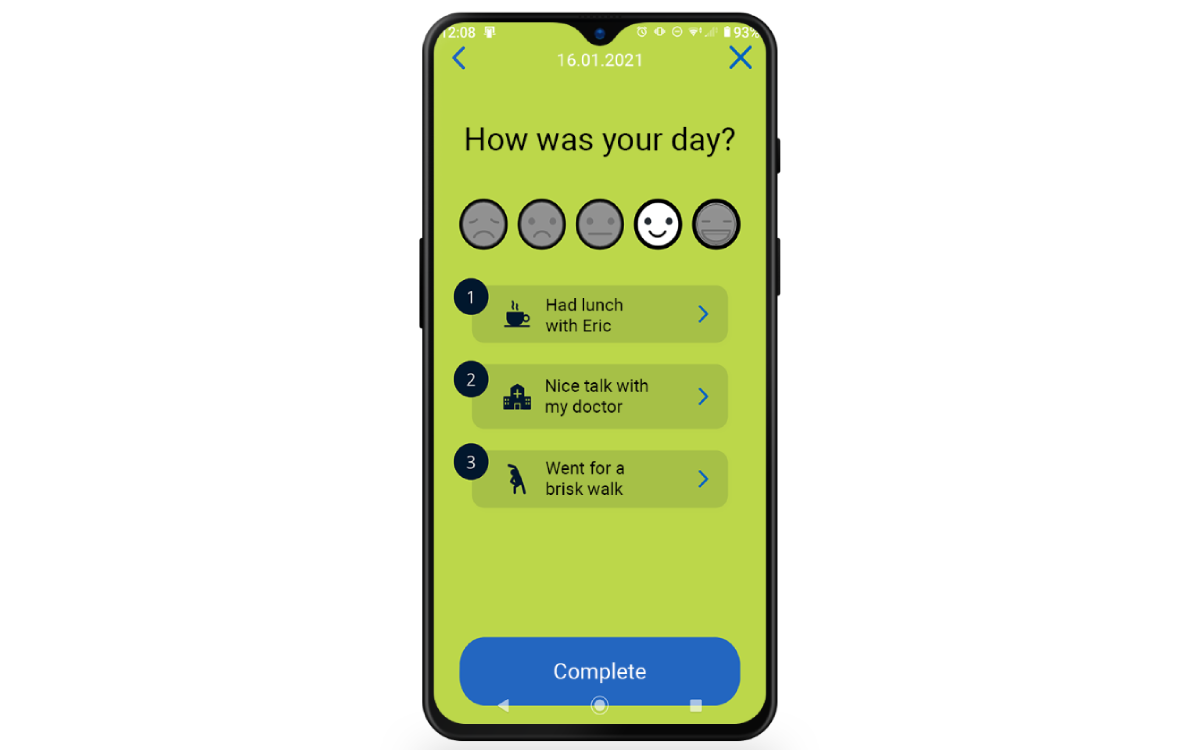
Smiley status and todays' three good things.

**Figure 6:**
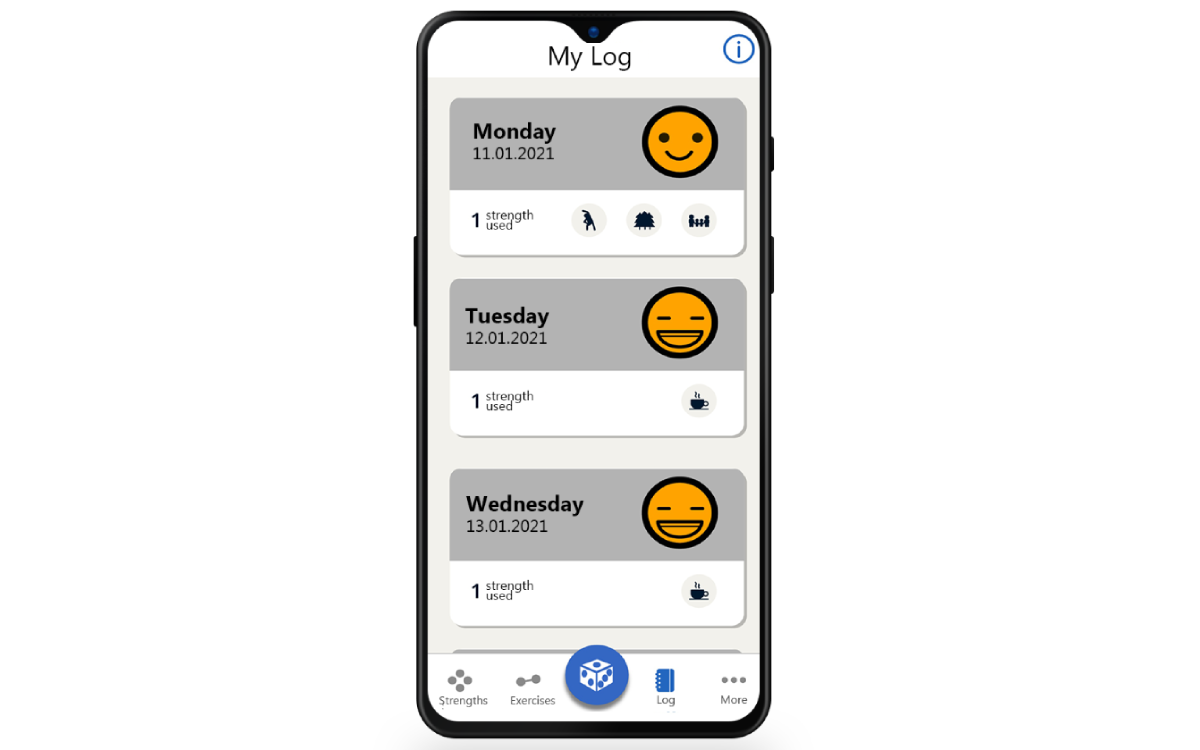
Log presentation.

Another key feature of the MyStrengths app is a logbook in which users can register how their day has been by choosing between 5 smiley faces ranging from sad to happy (see Figure 5). This logbook also asks the users to do the positive psychology exercise of entering three good things in life they experienced that day [7,58]. Through the presentation of all logged information, each day is summarized as cards with its “smiley status,” three good things, and strengths used (Figure 6).

**Figure 7:**
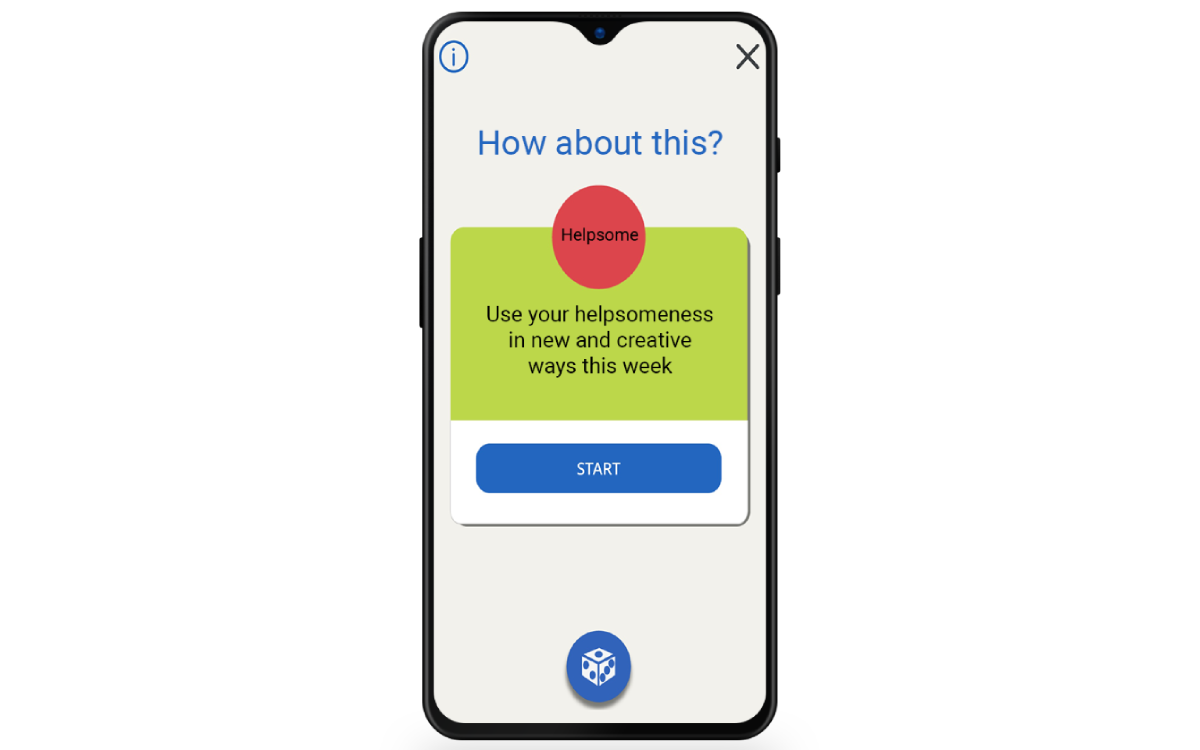
Die suggesting random exercise.

To instill a sense of playfulness and surprise, the menu panel of the app (as seen at the bottom of Figures 1, 6, and 7) also has a die. When the die is pressed, a random exercise will be suggested (Figure 7) for one of the strengths that the user has rated as either having or partially having.

### Aims

This study seeks to explore and evaluate ways of using mHealth to support people living with chronic illnesses in recognizing and using more of their personal strengths in their daily life to improve their overall well-being and quality of life. The study’s primary aim is to, through a month-long explorative feasibility trial, examine the study participants' impression of and experiences with strengths-focused mHealth apps in general and the MyStrengths app specifically. Given the wide range of measures used to examine outcomes or efficacies of strengths-focused interventions for the target group [24-30], a secondary aim of this explorative feasibility trial is to explore which of these measures, if any, might preliminarily indicate effects from the use of the MyStrengths tool.

## Methods

### Recruitment

The project was planned and conducted in adherence to the principles of the Declaration of Helsinki [59] and approved by the Privacy Protection and Data Security committee at Oslo University Hospital (project #18/05449). All participants or their legal guardians signed informed consents before taking part in the study. Each participant received a gift card valued at Norwegian kr 250 (approximately US $30) as compensation for participating.

Participants were recruited through multiple channels: through patient organizations or patient groups; through various health care providers, such as hospital educational centers and rehabilitation units; through advertising the trial on social media, such as the research team webpage and Facebook; and through our departments existing network.

Criteria for inclusion were being over the age of 16 years and speak Norwegian, having 1 or more chronic illnesses (self-report), and being smartphone user (either Android version 4.6 or newer, or iOS version 11 or newer).

### Study Design

Between November 2018 and March 2019, a 31-day feasibility trial was conducted using a mixed methods pretest-posttest design, consisting of outcome measures being completed before and after the trial period, logged data of participants’ app use (ie, system use), and optional interviews posttrial. Using a mixed methods approach is well suited for investigating possible effects, or indications thereof, from using the MyStrengths app while also exploring the individual participants' experiences and views [60,61]. Participants enrolled at their convenience during the trial period, and later, after 14 days of use (halfway through the trial period), the third author (EHB) called each participant and checked whether they had any issues or questions regarding the app or study.

### Data Types and Measures

To reach the study aims, a range of data types and measures were included.

#### Sociodemographic and Disease-Related Information

A study-specific questionnaire including questions related to age, gender, marital status, diagnosis, participant’s time living with illness, and level of experience with smartphones and tablets was collected at baseline.

#### System-Use Log

Data logging each user’s system use, with details related to app use, progress, and text input, were collected continuously, encrypted, and stored at the Services for Sensitive Data at the University of Oslo.

#### Psychosocial Outcome Measures

To achieve this study’s secondary aim—to explore whether outcome measures used in strengths interventions [24–30] can indicate effects from the use of the MyStrengths app—we employed the following outcome measures as part of the pretest and posttest (ie, at baseline and after completion of the 31-day trial period).

##### Strengths Use Scale

The Strengths Use Scale [62] is a 14-item inventory measuring awareness and use of own personal strengths. The items are scored on a 7-point Likert scale from strongly disagree to strongly agree. Higher scores indicate higher use of one’s strengths.

##### The Positive and Negative Affect Schedule

The Positive and Negative Affect Schedule [63] is a self-report questionnaire consisting of a 20-item scale describing the 2 main dimensions of people's mood: positive and negative affect. The items are scored on a 5-point Likert scale from very slightly or not at all to extremely. Higher positive affect indicates high energy and pleasurable engagement, and low positive affect indicates lethargy and sadness. High negative affect indicates distress or general unpleasurable engagements, whereas low negative affect indicates calmness and serenity [63].

##### Health-Related Quality of Life

The RAND-36 Measure of Health-Related Quality of Life [64] consists of 36 questions related to health-related quality of life, with 8 subscales (ie, physical functioning, role limitations caused by physical health problems, role limitations caused by emotional problems, social functioning, emotional well-being, energy/fatigue, pain, and general health perceptions). The response options are on various Likert scales (eg, 1-2, 1-5,1-7). The scale options ranges include “all the time to not at all,” “absolutely right to absolutely wrong,” “nothing to strongly,” and “excellent to bad.” Higher scores indicate better functioning.

##### Self-Efficacy

The General Self-Efficacy Scale [65] assesses the strength of a person’s belief in their ability to overcome challenges or respond to new and challenging situations. The scale consists of 10 statements, such as “I can solve most problems if I invest the necessary effort,” that are rated on a 4-point Likert scale, ranging from “not true at all” to “exactly true.” Higher scores indicate a higher degree of general self-efficacy.

##### The Center for Epidemiologic Studies Depression Scale

The Center for Epidemiologic Studies Depression Scale [66] measures depressive symptoms and contains 20 statements regarding the participants’ feelings over the past week, such as “I cried” or “I felt happy.” These are rated on a 4-point Likert scale ranging from “never” to “almost the entire time,” with higher scores indicating the presence of more symptomatology.

#### User Experience Measures

Good usability, user-friendliness, and engagement are essential to the success and adoption of mHealth tools [67]. To explore the participants’ experiences with the MyStrengths app, the study included 3 user experience measures in the posttrial test.

##### System Usability Scale

The System Usability Scale (SUS) [68] measures the users perceived usability of MyStrengths. The SUS is a 10-item questionnaire used as an end-of-test subjective assessment of a system’s overall usability [69]. Each item contains a statement (eg, “I found the system unnecessarily complex” or “I found the various functions in this system were well integrated”) that is scored on a 5-point Likert scale, ranging from “strongly disagree” to “strongly agree.” Higher scores indicate better usability.

##### Flow State Questionnaire

The “Flow State Questionnaire” of the Positive Psychology Lab [70] aims to measure the users' optimal experience or flow [67]; that is, the experienced absorption in the activity and whether the balance between challenges and skills is optimal. The questionnaire contains 20 items in the form of statements (eg, “I could effortlessly perform well” or “Time passed faster than I thought it did”) that are scored on a 5-point Likert scale ranging from “strongly agree” to “strongly disagree.” Higher scores indicate a higher level of flow.

##### Personal Involvement Inventory

Users’ involvement with mHealth tools has been shown to be related to their intrinsic motivation for using the tools [67,71]. To measure this, we used the shortened version of the Personal Involvement Inventory [72], a 10-item self-report measure gauging involvement and engagement in a tool or service. It contains 10 different statements, all beginning with “To me, the MyStrengths app is:” that are rated on a 7-point Likert scale with bipolar adjectives at the extremes, including “important” versus “unimportant,” or “boring” versus “interesting.” Higher scores indicate more involvement.

### Data Collection Procedures

The outcome measures and system-use log (app activity) were collected online through the secure Services for Sensitive Data at the University of Oslo, Norway. Participants received a link via email to access a secure web portal to access and complete the pre- and posttrial outcome measures. The pretest link was sent to participants as a baseline and completed before receiving access to the app. The posttest link was sent after the participants had completed the 31-day trial period. The system log data were encrypted by the app and then automatically sent to the secure Services for Sensitive Data.

After completing the posttest, all participants received a thank-you phone call from the research team, in which they were also invited to take part in a follow-up interview if interested.

The interviews were semistructured [73,74] and conducted by either the first (SJ) or third (EHB) author. Each interview lasted approximately 30 minutes and was guided by a semistructured interview guide covering 4 topics: (1) general impression(s), (2) strengths functionalities, (3) design, and (4) additional input and free feedback. The complete interview guide, including suggested questions and follow-up questions, translated to English, is available in Multimedia Appendix 1. To ensure the interviews were conducted similarly, the interviewers conducted the 2 first interviews together and subsequently made necessary adjustments to the interview guide. All interviews were audio-recorded, and the interviewer also took notes during the interviews.

### Analysis

#### System-Use Log

The system use log was transferred from the secure Services for Sensitive Data and imported into Microsoft Excel [75] as one large comma-separated values file. During cleaning and sorting, only data from users who completed both the pre- and posttest were kept. This decision was made to allow us to have parity in the data from the use-log and quantitative outcome measures. Log data for 31 days (ie, from the first login) were used and examined for each participant. Using Power Queries in Microsoft Excel, the use metrics’ final tallies were extracted.

#### Outcome Measures and Questionnaires

The outcome measures and questionnaires data were transferred from the secure Services for Sensitive Data and imported into Microsoft Excel as one spreadsheet. The data were cleaned and sorted by the first author (SJ), and, as with the system use log, only data from participants completing the trial were included. Only including data from users completing the trial allowed us to apply a repeated-measure design to analyze data, more easily identify any effects on the measures used, and facilitate having the same population for all the quantitative measures. The individual forms were imported into SPSS 26 (IBM Corp) [76], and the individual outcome measures’ scores were calculated following each measure’s specific guides and procedures. Pre- and posttest change scores were calculated, and their significance was tested using paired-sample *t* tests. Cohen *D* effect sizes [77] were also calculated for each measure.

#### Interviews

The interviews were analyzed using qualitative content analysis [78] following a selective coding strategy [73] that focused on the participants' use of and experiences with the MyStrengths app. The analysis was conducted by SJ and EHB, who also had conducted the interviews.

First, SJ and EHB read the transcribed interviews to familiarize themselves with the entire data corpus. The transcripts were imported into NVivo 12 (QSR International) [79], and, employing a directed approach [80] and using the main topics from the interview guide (ie, general, strengths, functionalities, design, and free feedback) as initial codes, SJ and EHB separately coded 2 interviews. The codes were then discussed between the coders and merged into a new list used to code 4 more interviews. The code list was then discussed between SJ, JM, and EHB, and further adjustments were made. The updated code list was then used to code all interviews. At this stage, the coding process and code list were also discussed with a colleague well experienced with qualitative methods who served as an auditor. Disagreements were discussed until one final set of coded material was reached. In the end, the coding process resulted in 5 top-level codes: (1) background, (2) use of the app, (3) design and functionalities, (4) usability and user-friendliness, and (5) experienced usefulness. Further, the coding yielded 21 subcodes in level 2 and, below these, another 13 in level 3. Examples of the code hierarchy included design and functionality → strengths and assessment → spheres or colors (design), background → strengths → awareness, and usability→ improvements → split up/hide/focus on strengths. Using the final code list, SJ and EHB authors coded the entire corpus. To illustrate the findings, quotes from participants are included throughout the presentation of the results. To ensure participants’ anonymity, quotations presented are not linked with diagnosis or demographic information.

## Results

### Participants

**Figure 8:**
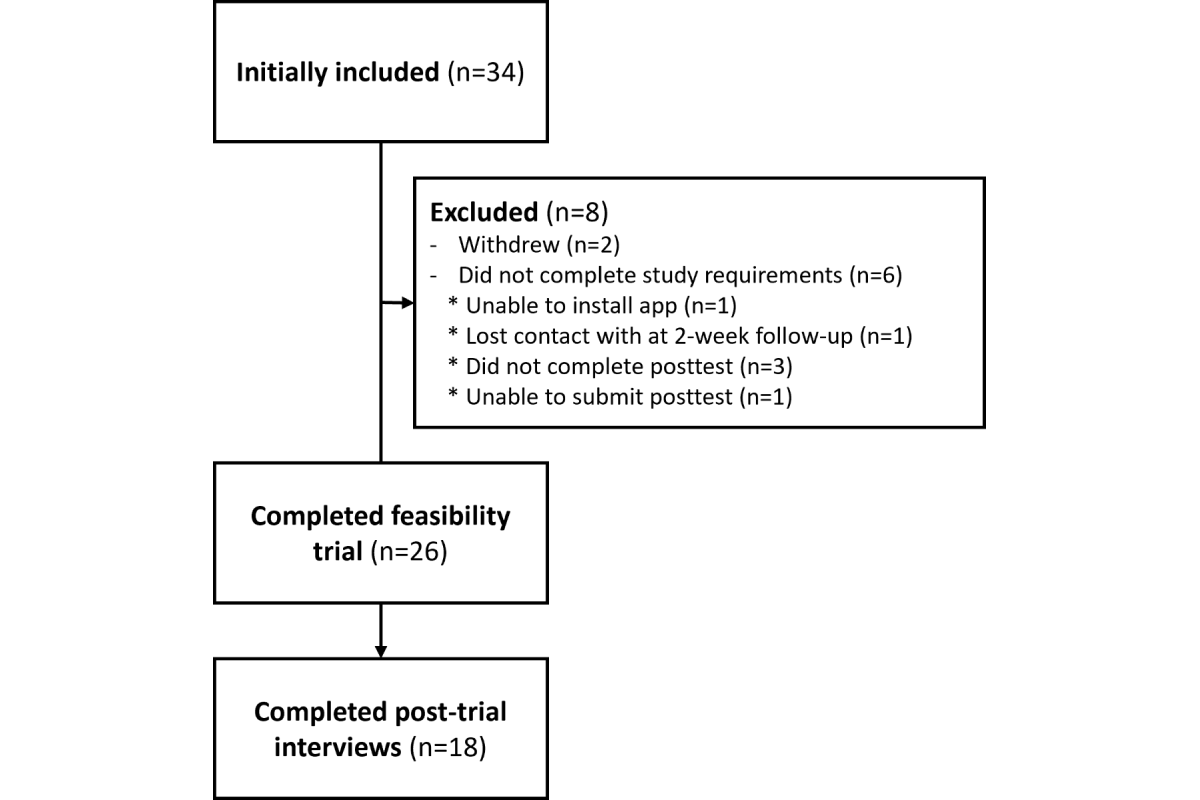
Recruitment and participant flow.

In total, 34 participants were initially included in the trial. Of these, 2 withdrew from the study, 1 could not be contacted and thus did not respond postbaseline, 3 could not be contacted and thus did not respond after the follow-up call at approximately 2 weeks, 1 was unable to install the app and therefore did not participate in the trial, and 1 had technical issues with the posttest and therefore did not complete the follow-up questionnaire. Consequently, 26 participants completed the trial, including the pre- and postmeasures. After trial completion, the participants were invited to a posttrial interview, in which 18 agreed to participate. Figure 8 presents the recruitment flow of the participants.

Demographic and background information for the 26 participants completing the feasibility trial is presented in Table 1.

There were few demographic differences between the completers and noncompleters: 1 of the 8 noncompleters was male, and they had an average age of 54 (range 37-61); 7 of 8 had a lot of experience with smartphones or tablets; and 6 of the 8 reported 7 (maximum) fondness for mobile or computer technology.

**Table 1:** Participants, characteristics, and demographic information (N=26).

Participant characteristic		Value
Age (years), mean (range)		46.8 (29-62)
Age (years), median		48
**Gender, n (%)**		
	Men	1 (3.8)
	Women	25 (96.2)
**Marital status, n (%)**		
	Married/cohabiting	18 (69.2)
	Single/divorced	8 (30.8)
**Educational level, n (%)**		
	Elementary/high school	9 (34.6)
	University < 4 years	14 (53.9)
	University > 4 Years	3 (11.5)
**Employment status, n (%)**		
	Full-time/part-time work	9 (34.6)
	Student	2 (7.7)
	Sick leave/disability benefits	15 (57.7)
**Diagnosis^a^, n (%)**		
	CFS^b^/ME^c^/fatigue/fibromyalgia	12 (46.2)
	Mental health	5 (19.2)
	Cancer/cancer related	3 (11.5)
	Rheumatic	5 (19.2)
	Chronic pain	8 (30.8)
	Neurological (eg, MS^d^)	6 (23.1)
	Other medical (eg, diabetes, kidney failure, Crohn)	8 (30.8)
**Time living with illness (years), n (%)**		
	0-5	9 (34.6)
	6-10	5 (19.2)
	11-20	6 (23.1)
	20+	5 (19.2)
	Did not respond	1 (3.8)
**Experience with smartphones/tablets, n (%)**		
	None	0 (0.0)
	A little	1 (3.8)
	Somewhat	6 (23.1)
	A lot	19 (73.1)
**Fondness for mobile/computer technology, n (%)**		
	1-2	0 (0.0)
	3-4	1 (3.8)
	5-6	10 (38.5)
	7	15 (57.7)

^a^Participants could report having more than 1 diagnosis.

^b^CFS: chronic fatigue syndrome.

^c^ME: myalgic encephalomyelitis.

^d^MS: multiple sclerosis.

### System-Use Log

**Figure 9:**
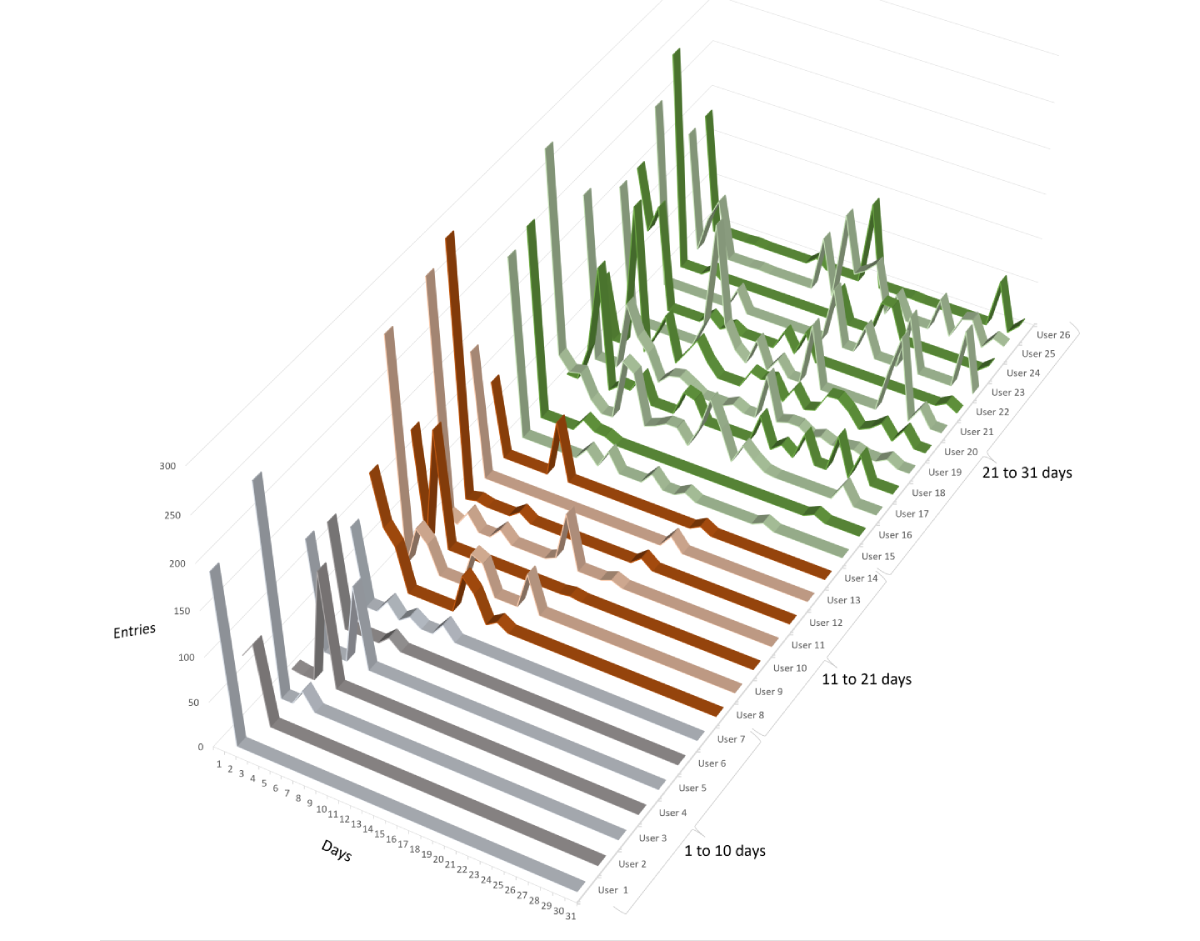
Data points per day per user. The x-axis displays each of the 31 days of the trial. The y-axis displays the number of registrations per day. The z-axis displays 1 lane each for the 26 users. The 3 colors are tiers of the last day with uses 1-10, 11-20, and 21-31, and with light and dark shades alternating for visual clarity.

The 26 participants logged a total of 8738 unique actions during the 31-day trial period, and 14 of the 26 participants used the app for a period (days between first and last registered activity) of 19 days or more. Of these, 11 used it over a period of 26 days or longer. Table 2 shows the distribution of the participants' use periods. On average, the users had 6 days with registered app activities. Figure 9 presents all 8738 logged data points from each user per day in the trial period (sorted after the last day of use). Although the users varied in both number of days with use and frequency, most activities took place early in the trial period, with as much as 45.65% (3989/8738) logged already on the first day of use.

**Table 2:** Use period (days between first and last registered app activity).

Period of use (days)	Participants, n (%), (N=26)
1	1/26 (4)
2-6	5/26 (19)
7-11	1/26 (4)
12-18	5/26 (19)
19-25	3/26 (12)
26-31	11/26 (42)

The statistics of use for the unique features of the MyStrengths app are presented in Table 3. The participants, on average, rated 37 strengths as “having” or “partially having,”. On average, only 8.6 strengths were rated as “not having.” Ten of the participants did not rate any strengths as “not having.” It should be noted that users could change the rating on their strengths, and thus some strengths are rated multiple times. Most of the participants (ie, 24) started at least 1 of the preprogrammed strengths exercises, and half of these also added their own exercise(s). Sixteen participants added items to the “three good things in life” activity, which they did an average 8.9 times. The die, which suggests strengths exercises from strengths rated by users as “having” or “partially having,” was used by 23 participants, who in total started 86 suggested strengths exercises.

**Table 3:** Features and use statistics (N=26).

Feature		Participant use, n (%)	Entries		
			Number	Mean	Median
**Strengths**					
	Strengths rated total	26 (100)	1122	43.2	37.5
	Strengths rated as “Having”	26 (100)	584	22.5	20.0
	Strengths rated as “Partially Having”	25 (96.2)	362	14.5	13.0
	Strengths rated as “Not Having”	16 (61.5)	138	8.6	4.5
	Strengths skipped/not rated	5 (19.2)	38	7.6	9.0
	Strengths reflection added (free text)	17 (65.4)	101	5.9	3.0
	Custom strengths added (free text)	2 (7.7)	3	1.5	1.5
**Strengths** **exercises**					
	Exercises started	24 (92.3)	192	8.0	5.0
	Exercise rated	19 (73.1)	139	7.3	5.0
	Custom exercises added (free text)	12 (46.2)	44	3.7	3.0
	Exercise comment added (free text)	15 (57.7)	84	5.6	3.0
**Daily log**					
	Three good things in life added (free text)	16 (61.5)	143	8.9	5.0
	Daily log smiley face registrations	21 (80.8)	100	4.8	3.0
**Die**					
	Die menu opened	23 (88.5)	83	3.6	2.0
	Suggested exercise started	20 (76.9)	86	4.3	2.0

### Free-Text Entries

The MyStrengths app allows users to add comment or free text of up to 500 characters at various points in the app. The logging also recorded these. An overview of these types of entries is presented in the following sections.

#### Strengths and Strengths Reflections

In total, 17 of the 26 users added 101 free-text entries as reflections on their strengths. These entries ranged from single word affirmations that they had this strength (eg, “yes”*)* to summaries of activities or situations they had taken part in (eg, *“*played with the children today,*” “*getting better at this*”*). Some comments were celebratory and regretful at the same time. For instance, connected to the strength “I know how to set limitations for myself,” a user wrote, “I have gotten better at setting limits for myself, but many others do not understand this.*”* Some of the reflections also had deeper, personal content. For instance, as a reflection on their strengths, one person wrote the following:

I appreciate what is good in life. After my child died [as a teenager], life has morphed into something different. Still, I have a good life today, 5 years after.

A similar comment to the strength “I am at peace with my situation” came from another participant who wrote the following:

When my daughter died [some years ago], it was nice to be able to come to peace with the situation.

Although 17 of the participants added reflections to their strengths in the app, only 2 added a total of 3 new strengths into the app (ie, “I am open and share with others,” “I am efficient,” and “I am fond of animals”).

#### Strengths Exercises

Eighty-four free-text comments were added to the strengths exercises. Some were simple congratulatory comments participants included for themselves, such as “great” and “works OK, and is important,” or brief reflections, such as “good feeling both physically and mentally,” or “gives me strength and confidence.” Some of the comments were also more comprehensive and contemplative, as exemplified by these two quotes:

Workout! Even if it’s only taking a walk around the area. Time to myself is so important and shall be prioritized for my own, as well as my family’s sake.

Participants added a total of 44 custom (or adapted) strengths exercises in the app. Most of these were simple and primarily specified activities the users planned to do, such as “take a walk,” “rest enough,” “meet NNN once a month,” “Yoga,” or “change medication dose.” As with the strengths reflections above, a few of these inputs were also of a more cognitive reflective nature, such as the following: “appreciate the little things in life: coffee, smells…”, “grow when facing obstacles, find people with knowledge, ask for help”, and “thank others for being good people in my life.”

#### Daily Log

In the daily log, 143 entries were added as “good things” of the day. Roughly half of these were brief, such as “worked 8 hours today” and “went for a walk.” The rest were more elaborate, for instance, the following:

Did a walk with dad, although I am not feeling great. Perfect weather!

as able to take breaks instead of pushing on. A bit drowsy and unwell today, so happy with that.

A few of the daily log comments were longer and reflective as well as personal, such as the following:

Been very tired the past few days, with lots of pain in the neck. Today started badly with a foul mood and arguing with the kids, and it gets worse for me when I am so frail. My daughter went off sad, returned, and gave me a long hug before properly saying goodbye and leaving for school. That felt very good!<3

### User Experience Measures

#### System Usability Scale

The system usability was rated as 61.5, which is considered a below-average experience. The center of the scale has been rated at 68, and the de facto industry standard for providing an above-average user experience is to achieve 80 points [81]. This points to the overall user-friendliness of MyStrengths being less than adequate.

#### Flow State Questionnaire

The main score was 2.83, which is slightly above the scale midpoint of 2.5. Considering the 2 subscales separately, the balance (task complexity) subscale averaged 2.58, and the absorption subscale averaged 2.90. This indicates that the overall flow intensity was medium, with the participants rating their engagement (absorption) with the tool as somewhat higher than that of the balance between task complexity and their skills.

#### Personal Involvement Inventory

The score was 3.85, which is slightly (0.35 points) above the scale’s midpoint, pointing to an only slightly above medium involvement. This likely indicates that the participants did not find MyStrengths either fascinating, exciting, or appealing; or mundane, unexciting, or unappealing; but instead somewhere quite in between.

#### Psychosocial Outcome Measures

The pre- and posttrial outcome measures yielded effect sizes, Cohen *D* [77], mostly in the small to medium range. The paired-sampled *t* tests only yielded significant changes in the general health dimension of the RAND-36 Measure of Health-Related Quality of Life. The results from the statistical tests and calculations are presented in Table 4.

**Table 4:** Statistical results from the pre- and posttests.

Measure		Mean pretest (n=26)	Mean posttest (n=26)	Mean difference	SD	SE	*t* value	*P* value	Effect size (Cohen *D*)
Self-efficacy^a^		2.82	2.95	0.13	0.43	0.08	1.56	.13	0.31
**PANAS^b^**									
	Positive affect	24.12	23.65	–0.46	4.12	0.81	–0.57	.57	–0.11
	Negative affect	22.31	22.23	–0.08	6.03	1.18	–0.07	.95	–0.01
CES-D^c^		21.12	19.12	–2.00	7.12	1.40	–1.43	.16	–0.28
Strengths use^d^		4.29	4.49	0.21	1.01	0.20	1.04	.31	0.20
**Health-related quality of life^e^**									
	Physical function	59.23	60.38	1.15	18.13	3.56	0.33	.75	0.06
	Social function	41.35	41.35	0.00	13.69	2.69	0.00	.99	0.00
	Role physical	12.50	19.23	6.73	26.98	5.29	1.27	.22	0.25
	Role emotional	75.64	70.51	–5.13	45.89	9.00	–0.57	.57	–0.11
	Mental	71.85	73.23	1.38	7.83	1.54	0.90	.38	0.18
	Vitality	26.15	28.27	2.12	14.57	2.86	0.74	.47	0.15
	Pain	36.73	37.36	0.63	21.86	4.29	0.15	.89	0.03
	General health	31.15	37.31	6.15	14.02	2.75	2.24	.03	0.44

^a^General Self-Efficacy Scale.

^b^PANAS: Positive and Negative Affect Schedule.

^c^CES-D: Center for Epidemiologic Studies Depression scale.

^d^General Strengths Use Scale.

^e^RAND-36 Measure of Health-Related Quality of Life.

### Interviews

The interviews covered many facets of the use of the MyStrengths app as well as of life living with chronic illness in general, and data material coding and analysis identified 4 major topics: strengths and overall thoughts on MyStrengths, features and functionalities, design and usability, and the strengths approach. These main topics are presented in the following sections.

#### Strengths and Overall Thoughts on MyStrengths

##### Familiarity with Strengths

Most participants reported having some familiarity with thinking about their strengths, and only 2 participants stated that this was a new concept to them. When describing their connection to strengths, 1 participant, who had been ill for many years, described her strengths focus as a natural part of her daily life:

… That is what I feel I have been good at over the years, thinking of my strengths—and using them.

Several interviewees reported having become familiar with thinking of their strengths through courses and rehabilitation activities they had attended over the years. When comparing the MyStrengths app to previous experiences with strengths-thinking, one participant said the following:

Through the years, and this is the ninth time I am at physical rehabilitation, we have tried to focus on… on these things. But, it has always been—you know, together with others, so it is really nice to be able to do it at home too.

##### Perceived Usefulness of MyStrengths

In terms of their perceived usefulness of the MyStrengths app, the interviewed participants were evenly split 3 ways.

First, 5 of the interviewed participants that completed the trial reported finding the MyStrengths app useful and described enjoying the app and its novelty. Some of these users were new to the strengths concepts and appreciated the newly raised awareness about strengths, while others reported already being aware of some of their strengths yet found the strengths exercises beneficial. In describing the benefits of the MyStrengths app, one user succinctly summarized her experience:

…I feel like I have become more aware of my strengths, and that I have used them more consciously, and this has already led to… well actually positive changes in my life… I thought I already knew this, but it is something about using it yourself and becoming more aware.

Five of the interviewed participants found the app to be somewhat useful, both liking and disliking various aspects of it and describing the design and the content to be adequate. One participant stated the following:

It was a couple of times when I kind of went “oh yeah, that is right, things are quite all right with those things, and those.” And I thought that was nice. But, it was not really revolutionary in any way.

Finally, 6 did not find the app useful. Reasons ranged from already being familiar with ones’ strengths, favoring a more straightforward, to-the-point solution if in app form, or preferring the content be delivered as courses. Another repeated response from these users was that MyStrengths was “too comprehensive” and “demanding” due to many questions, prompts, or requirements for reflection.

#### Features and Functionalities Of MyStrengths

##### Strengths Assessment

The predefined list of strengths in the MyStrengths app contains 40 items. The participants, on average, rated more than half the strengths as “having,” and during the interviews, participants stated that seeing their strengths motivated them, gave them something to be proud of, or reminded them of, for instance, of the positive things in their life. No interviewees reported missing strengths outside the 40 included items, and most reported no issues with seeing strengths that did not apply to them, with several also describing it as virtually impossible to make a tool that fits every user perfectly. However, some did describe finding the number of predefined strengths somewhat overwhelming. Some also found it challenging to assess the 40 items in one session and often split the assessment over multiple days: “I split it over 2 days…, it would have been too much to answer all in one go.*”* Another user, who also thought the number of strengths was high, would have preferred to limit the number of strengths visible at one time in the app:

I thought there were quite a lot of them, yeah. And it kind of became too many for me to go into each of them and... think it over... actually. So, had it been fewer, it might have been easier for me to use.

##### Strengths Exercises

The majority of participants had started at least 1 strengths exercise in the app. Many of the participants interviewed described having enjoyed the concept of strengths exercises, and one participant voiced a common sentiment in describing these:

…Obviously, it feels a bit more committing to fulfill what you have written down... I mean, it feels like something you must do as, well, one has promised to do it either to oneself or… yeah…

Several of the participants wanted more strengths-exercises to, in the words of one participant, “make it more interesting for long term use.”

The users varied in their appreciation of the different types of exercises. On average, the interviewees seemed to prefer physically oriented strengths exercises (eg, “aim to do something new this week,” or “show someone you care about that they are important to you, tell them why”), over the more cognitively oriented ones (eg, “think of one of your goals and break it down into smaller actions” or “take a few minutes and consider what it means to you to live in a safe environment”). Some of the approximately 80 suggested strengths exercises in the app were experienced as repetitions of things the participants described having done before in other settings or courses. A few also reported that the exercises could be either too time or resource intensive, and some described this part of the app as a bit overwhelming or too complex. Nonetheless, several interviewees also noted that the strengths exercises could help them better understand the connected strengths and their relevance to them.

##### Daily Log and Three Good Things Exercise

Sixteen of the participants added items to the “three good things” exercise, and many were already familiar with the exercise. Of the participants interviewed, everyone reported appreciating this exercise, particularly how it could help them get an overview of that day and emphasize their successes.

That is something I think is really nice, and I have good experiences with… to write down 3 good things and look back on that day, to see that I have actually mastered something. That is good to keep in mind and can be really uplifting and supportive.

Some additionally suggested having a more extensive range of icons to select from within this exercise and using prompts or notifications to remind them to do the registration daily or point out good things they had already registered.

##### Die feature

Most of the interviewed participants had used the die menu at least once, and many described the die both as a useful and engaging feature. These participants reported liking that it was a straightforward way to access the strengths exercises and that it would suggest strengths exercises they would not necessarily choose themselves. One participant, for example, likened the die to being challenged by other people:

I found it fun to press that [the die], and then see what would show up and whether it is something I could manage. Obviously, you could not do everything then and there. But it is nice to… well, I like to kind of, be challenged by someone, and not come up with everything myself, because then I tend to choose a bit easier and safer, I dare say, if I were to choose for myself.

A number of the interviewed participants also noted the engaging factor of using the die. Both in terms of how it provided surprises and how it was interesting to browse through the various strengths exercises presented.

I especially liked the dice… how you just can press it and then «ahh», maybe I will do that, and then I opt it out, maybe not that one… And there are so many small exercises or tasks. I think, I liked that there are so many suggestions…

#### Design and Usability

##### General Design

A number of those interviewed reported they liked the overall design of the MyStrengths app. Of the 18 interviewees. 9 generally liked the design, even though the degree of enthusiasm varied. For example, one participant stated the following:

Well, I think it was really nice. But… you know, nothing either “wow” or “non-wow.”

Another said the following:

I really like that [the MyStrengths app] quite well. It is… as we talked about, it seems a bit more playful, instead of a list and then bang, bang, bang—this is what you have to do. That is, like, very structured. I like that… with the spheres and that they float around; you kind of get the impression that it is not that serious but at the same time useful. And concerning, again back to mastery, you get a bit more pleasure out of working on these things.

Of the other half of the interviewed participants, 3 reported liking the design somewhat, and 6 reported not appreciating the app’s overall design, describing it as sometimes chaotic or cumbersome to use or sometimes being cognitively challenging.

##### Spheres

Approximately half of the interviewed participants reported liking the spheres, with one participant saying the following:

[It’s] a lot funnier, haha. Of course! I am like a small child who can press a button, and then things are colorful.

Some described the spheres as reminding them of a lava lamp, making the app appear somewhat “fresh” and calming. Others did, however, describe the spheres as somewhat confusing and difficult to track. Most participants reported appreciating the movement(s) of the strengths spheres, stating that movement made the app a bit more “alive.” At the same time, some participants also expressed some hesitance toward the movements, with one stating the following:

On a very tough day, it is tiring, but that is my personal opinion. Others might simply find it pleasant to see something with colors move slow, slowly...

Another user felt the same concern using the app on bad days but described being selective about which days she used MyStrengths, and stated the following:

I found it fun, haha… But, if I had a bad day, I might have gotten tired by it. Still, for the days I used it, it thought it was fine.

##### User-friendliness

The interviewed participants were also split related to the perceived user-friendliness of the MyStrengths app, with approximately half of the participants describing the app as user-friendly. For instance, these users remarked on how the colored spheres made them easy to distinguish by rating, how few steps were needed to access the strengths exercises (either through a specific strength or even easier by using the die), or that the limited set of features made it easy to use and navigate. Common issues raised by those who were less favorable were that the spheres changed places on the screen when moving between menus, they felt unsure as to what to do, and that it was easy to lose track of where they were in the app. Several participants suggested splitting the personal strengths assessment into multiple sessions or allowing the users to choose which spheres to keep active and onscreen.

Other comments related to use and usability included making MyStrengths available on larger devices such as tablets and PCs, using different colors on the spheres, or making the app more responsive. Lastly, some interviewees suggested the app could provide feedback on the actions done, such as congratulating them on completing an exercise, giving summaries of the strengths used in the past week, or, as mentioned earlier, adding options for notifications and reminders:

It had been super nice if you suddenly got a message which said “remember how good syou are at this” kind of. Then I would go “tnaaw,” how nice… I would like that a lot.

#### The Strengths Approach

In discussing participants’ experience with MyStrengths, the interviews also broached topics that touch on the overall subject of strengths-based approaches.

##### Vulnerability and Ambivalence

Most participants reported that using their strengths more actively resulted in positive feelings such as mastery or confidence. As one participant stated the following:

I think it creates a positive awareness without… talking of that app, without it reminding me of… I am bad at doing this, in a way—and that you can only get better at it.

However, some of the participants described a sense of ambivalence concerning specific strengths. For instance, strengths or even strengths exercises that a person had or could do before becoming ill might now raise a sense of, or trigger, sadness. Also, some of the strengths listed in MyStrengths could be considered external, focusing on users’ surroundings, such as family members, care providers, or economy, something that the users may have little control over. Discussing this, one participant stated the following:

I did find some of the items [in the strengths assessment list] to be a bit, a bit touchy. For instance, the ones with “those [people] around you”; you know, it is about people close to you and how things are in your surroundings. And for instance, when you are ill for a long time, a lot of that gets lost… I mean, it is nice to look and reflect on those things, but at the same time, it is a bit… wow, cannot do anything about that. I would like to, but I cannot. Just does not work.

Strengths lost were not necessarily considered a negative thing, however, and some also considered these as regainable and reasonable goals for things to do in the future. When discussing working with strengths assessed as lacking, one participant said the following:

It is a bit mixed. But, obviously, there are strengths assessed as “not having” that one actually had before becoming ill and that you would rather have back. But, you also know that you will not manage that today. And then it’s okay to focus a bit on that. Even though you cannot do anything about it at the moment, and you would have to have it as a goal to do something about it in the long run.

##### Exercising Strengths or Getting Rid Of Weaknesses?

When speaking of their strengths, many interviewees described these not only as positive attributes but as a concept existing in a dichotomous relationship with what they variably termed as weakness, faults, or errors.

Yeah, it was really that... or should I say, that you have somewhere you can go, to see how your day is working, or not, and to focus on yourself and your own stronger and weaker sides.

Most participants reported understanding and appreciating the concept of working on their strengths instead of their nonstrengths. Nevertheless, several reported also wishing to work on gaining strengths they perceived themselves as lacking. One participant, who was well experienced with mastery and strengths training, explained as follows:

I get happy when opening the app. It, it reminds me of what I manage and can. So yeah, there were new… I did actually get new tips through the exercises, things I was not aware of […] What is nice with it is seeing all the strengths, that makes me happy, everything that’s red [talking of the spheres rated as “having”], it makes you kind of want to go through the others [strengths], and get everything else red too

…

However, not all participants sought to gain new strengths, and some chose to focus on the strengths they had, as one stated the following:

Well, it was very few spheres that did not turn red [marked as having] … And of course, I feel I benefit from using and simply knowing that I am using, and being aware that when I am doing this, it is good for me. I am using some of my positive traits when doing this, and I think that is positive.

### Future Use?

When asked about whether they would keep using the MyStrengths app after the trial, the participants were, again, roughly split into 3 groups, with 6 stating that they would, 4 stating they might, and 5 stating they would not continue using MyStrengths (3 interviewees did not answer this question). Reasons for wanting to continue using the app included finding benefits from using it, such as the app being a reminder of what they are able to achieve, emphasizing what they can manage in a situation when things do not always go as planned, creating awareness of their strengths without also making them think of the weaknesses, and facilitating a more deliberate use of their strengths which has led to positive changes. Several participants also noted how the app could motivate and support them to do tasks and activities that they would not necessarily be able to follow through with on their own:

When you can put those tasks and exercises into the app… To me, living alone, it feels like more of a commitment when I have added it as something I shall do. It is motivating to know I have it there and that I can do something about it.

Among those unsure whether to keep using MyStrengths, several wanted to wait and see if new content or features, such as reminders and notifications, could be added. Participants could be put into 3 broad categories in terms of reasons for not wanting to keep using the app: (1) those who did not appreciate the overall design and visual concept, (2) those who did not find much use for the strengths exercises, and (3) those who reported already being quite familiar with their strengths. It should be noted, however, that even those in this last group found strengths that were new to them: “There was incredibly many there, and even some I did not even think of as strengths.”

### The Right Target Group

Regardless of what they thought about the MyStrengths app, all of the interviewed participants described appreciating the strengths approach, considered the strengths focus in chronic illness care to be an important one, and welcomed the MyStrengths app. One participant, a professional health care provider, would also recommend the app for people in her care:

I look forward to this, hopefully, being generally available for people with chronic illnesses so that I, as a professional, can recommend it. That would be really helpful.

Others reporting appreciating the MyStrengths app stated that it helped raise or increase awareness of their strengths. For instance, one participant stated the following:

Never done anything similar before. The first times I found it extremely hard… to see strengths... and after a while, you recognize it a bit more, that there are strengths, and you use them in this way… Sometimes, when not using the app, I think to myself, that is a strength you have used—and give myself a little pat on the shoulder.

However, several of those not describing the app as useful pointed out that even though they did not find it useful, the app could still benefit other groups of users. One interviewee suggested the following:

As I say, it is a very nice app, even though it did not really hit “bullseye” with me, and that some of the themes were a bit iffy… It is likely a much better fit for people who have not been “in the game” [of chronic illness] for as long as I have.

As such, it is possible that finding the right target group, rather than aiming for a “one-size-fits-all” approach, should be further explored.

## Discussion

### Principal Findings

Strengths interventions have been around for a long time, and as reported, many of the participants in this study had previously been introduced to strengths approaches through rehabilitation and educational courses. However, strengths-focused self-management support tools have not been available in the form of an app people can install on their phones. With MyStrengths, users can have access to strengths support right there and then. All participants interviewed in this study voiced support for strengths-based tools and provided a range of positive and negative feedback related to MyStrengths’ design and implementation. As there is little literature regarding strengths-focused mHealth tools, this trial has functioned both as an evaluation of the specific implementation, the MyStrengths app, and an exploration into the feasibility of strengths-focused mHealth interventions in general.

This study suggests that tools that focus on eliciting and mobilizing personal strengths, such as the MyStrengths app, may have the potential to support people living with chronic illness and to identify and use their strengths in their day-to-day life. The findings revealed 3 main points, which will be discussed in the following sections: (1) all participants could easily recognize multiple strengths within themselves; (2) the participants highly varied in their preferences for strengths exercises that they wish to practice; and (3) gameful designs can engage users, even when done in a soft and toned-down approach (ie, not emphasizing competitiveness or achievements).

### Strengths Interventions

#### Everyone Has Strengths

On average, the participants rated more than half of the 40 strengths during the assessment in the MyStrengths app as “having” and under 9 as “not having.” This is in congruence with one of the foundational underpinnings of the strengths concept: that everyone has strengths [7,13,82]. The findings also show that in a process similar to strengths nomination [11]*,* the users were able to assess their strengths using the MyStrengths app.

Although the participants all reported a substantial number of strengths, the collected data did not identify the time it took users to find their first strength. Niemiec [11] has discussed the sometimes tricky task of getting users to recognize their first strength when new to strengths interventions. However, this may have a cascading effect, and identifying more strengths can subsequently become easier, thus highlighting the importance of making sure users can easily recognize their first strengths. During the development of the strengths list used in the MyStrengths app [12], prospective users contributed greatly to the process of nominating and formulating relatable strengths items. Based on the sheer number of strengths the individual users recognized from this list in the current study, participation of prospective users in these phases may be highly beneficial.

Some of the participants also highlighted feeling a sense of vulnerability when assessing strengths they experience as weakened or lost by changing life circumstances. Moreover, as the strengths assessment presents strengths at random, one might encounter situations where users start by rating many strengths as “not having” or are reminded of strengths that are now weakened or lost. Providing a positive onboarding experience that introduces and prepares the users for the rest of the app can be crucial, and starting this by not recognizing many strengths is likely counterproductive. Ways to optimize the start of the assessment can therefore be to make sure the first few items presented during strengths assessment, for instance, are topically distinct (eg, being outgoing and social, having a supportive family, or being persistent), among the more popular strengths in the user group (ie, something that would need to be based on empirical evidence), or strengths that have been shown to be connected to life satisfaction (eg, love, gratitude, or zest) [83].

#### Internal and External Strengths

Typically, strengths tools and interventions are based around strengths that are internal to its users (eg, having zest, humility, or perseverance) [9,11]. However, MyStrengths also include external strengths and resources (ie, good relationships with health care providers, a supportive family, or living in a safe environment). The inclusion of these strengths stems from an earlier project by our research department [12], in which multiple external strengths were reported to be important by the participants. In the current study, however, several participants reacted negatively to some of these strengths, such as those related to caregivers or their economic situation. Some explained that these types of strengths are out of their control and are thus elements they cannot impact or work on acquiring. Addressing this type of issue, Kristjánsdóttir et al [84] grouped strengths into 4 categories (ie, personal strengths, strategies, resources in the environment, joy, and meaning), giving the users the choice of which category of strengths to assess. For MyStrengths, it might be that giving users the option to hide unwanted strengths could reduce the negative experiences from being reminded of strengths that are, for instance, out of reach.

#### Strengths or Weaknesses

Several interviewees reported wanting to work on what they considered their weaknesses, to regain strengths that had been weakened with their illness, or to simply gain as many strengths as possible instead of focusing on actually using their strengths. The benefits of the strengths approach come not only from knowing one’s strengths or by having as many as possible but also from using them actively [11,13,83], and while working on one’s strengths can lead to further growth, remedying one’s deficiencies merely returns the person to a state of equilibrium [13]. Nonetheless, the wish for working on both strengths having and not having was often voiced by participants during the development of the MyStrengths app [23]. Therefore, the home screen was designed to give users an overview of all strength items rated, including those rated as “not having.” To place primacy on the users’ present strengths, however, the spheres are automatically sorted, and those rated as having are prioritized and shown above those either partially or not having. The broaden-and-build theory [85,86] posits that everyday positive emotions can contribute to a cascade of other positive emotions. Thus, if users can get a positive experience from mastering new things, such as working on strengths they do not (yet) possess, this might also contribute positively to the overall experience of the app. Additionally, allowing users to work on the strengths of their choice can also increase their sense of autonomy, contributing to motivation and overall positive experiences [87].

Strengths interventions are typically conducted face-to-face [11], affording the therapist or counselor to continuously adapt the session to the receiver. With mHealth tools, this type of guidance is usually not available, and making sure introductions and tutorials more than adequately cover the purpose and rationale of the strengths-based approach should be of high importance. The presented findings indicate that providing just a short introduction when starting the app for the first time was insufficient. Therefore, more comprehensive approaches, such as giving users an in-person introduction where the app, its content, and rationale is presented and discussed in more detail may be beneficial [43].

#### Strengths Exercises

The feedback on the MyStrengths strengths exercises was mixed, and preferences for types of exercise seemingly depend on personal taste. Børøsund and colleagues [43] suggested that variation in exercises and exercise types is beneficial to support use. Similarly, some users in this study appeared to prefer exercises asking them to do something concrete, while others appreciated the more cognitively oriented exercises. A multitude of sources for strengths exercises and activities exist [7,11,33,38], yet little has been published on the actual development of such exercises and activities. Even though based on or strongly inspired by exercises described by existing literature, the exercises used in MyStrengths have been adapted to fit the connected strength. Although user evaluations have been conducted for these exercises [23], further improvement can be gained from the input provided by participants in our study. Additionally, the free-text inputs in the app during this trial yielded valuable insights into how users themselves write strengths exercises.

Based on the variety of individual tastes and preferences related to strengths, future strengths-focused tools should include multiple varied exercises or activities related to each strength. The pool of exercises could further be expanded by allowing users to share exercises they have created with other users. These two points could potentially increase the likelihood of users finding content to their liking and could also carry the additional benefit of users not having to repeat exercises but instead find new content for a more extended period of using the app.

#### The Right Target Group?

Regardless of what they thought about the MyStrengths app, all interviewed participants described appreciating the strengths approach and considered the strengths of chronic illness care to be important. However, those not finding the MyStrengths app useful did comment that it might be better suited for people relatively new to living with their health issues. This also reflects user feedback received throughout the development of MyStrengths [23], and future research should consider including more participants “new” to living with chronic illness when designing non–disease-specific mHealth support and educational tools.

### Design and Usability

#### A Toned-down Gameful Design Approach

As mHealth tools naturally lack the person-to-person connection common when seeing a counselor or a physiotherapist, it is essential to design such tools in manners that increase the likelihood of them being used as intended [88]. With this in mind, gameful designs have become a common design approach for mHealth apps specifically [50,54] as well as for apps in general [89]. Design features that are competitive or otherwise highlight the user’s performance, such as the number of strengths assessed or exercises completed, have nevertheless been avoided in the MyStrengths app. The reasoning for this approach is that, as per the strengths perspective, the focus should be on using one’s strengths, irrespective of how many there are, and not necessarily focusing on identifying and adding new ones [11,13]. Another critical aspect supporting this choice is that guidelines [90] and empirical studies [91] have cautioned against relying heavily on competitive elements in mental health and well-being interventions.

User engagement can also be encouraged through softer, more toned-down, gameful design approaches, such as creating user experiences that are visually pleasing and enjoyable [50]. Examples of this can include theming the app as a growing garden, in which growth is a metaphor for one’s progression and development [91,92] or using the metaphor of a journey toward a more flourishing life [49]. Using these kinds of metaphors was also suggested by participants in the co-design activities during the early development of MyStrengths [56]. The home screen of the MyStrengths app draws inspiration from the ubiquitous lava lamp (ie, a bottle-shaped lamp in which colored shapes of molten wax slowly floats around) and is designed to encourage engagement by presenting the users’ strengths in a playful and calming manner. Although interviewees in this study were split in their appreciation of this design concept, those in favor reported enjoying the colors and the movement of the spheres on the home screen.

One archetypical game design element that holds potential is randomness [93]. However, it has not been widely used within mHealth tools for well-being or mental health [94]. In MyStrengths, the die draws exercises at random, and many users reported liking this feature and used it to find exercises to their liking quickly. By throwing the die themselves, users choose to “ask” for exercises, which may also aid users in maintaining autonomy instead of the app unsolicited suggesting exercises. The die suggestions further seem to create similar experiences to, or function as a substitute for, social interaction. As voiced by several participants, the exercises suggested by the die were acted upon much in the same way as if they had come from other people. Thus, although the MyStrengths app does not include any social features, it still appears to have provided users with some form of social motivation, which can be very powerful [87,95]. Despite the seeming ubiquity of social features in mobile apps, none of the participants interviewed reported missing social features such as sharing, collaboration, or communication in MyStrengths.

#### Use and User-friendliness

The home screen is almost exclusively focused on the strengths and the assessment of strengths, while all other features are available after navigating to other screens in the app from the menu at the bottom of the screen (Figure 1). Sieverink et al [96] reported that users largely followed the structure and paths of use presented on the main screen. It might thus be that the focus on strengths and strengths assessment partially can explain the vast difference in use between the assessment and the other features (see Table 3). For instance, 1122 strengths were rated, while only 192 exercises were started, or 143 “three good things in life” were added. On average, the participants used MyStrengths on 6 unique days during the trial period, and 45.65% (3989/8738) of the total logged activity was done on the first day of use alone. As presented in Figure 9 users stopped using the app steadily throughout the trial period. A decline in use over time is, however, typical in eHealth and mHealth [97]. In their study of a mHealth tool for diabetes management, Adu and Colleagues [98] found that use steeply declined when reminders were disabled after 2 of the 3 weeks of the trial period. As also supported by participants during the interviews, it is likely that notifications or reminders from the MyStrengths apps could thus have contributed to maintaining user engagement both in terms of duration and frequency of use.

When creating mHealth tools with gameful approaches, it is also essential to make sure such design choices do not make the tools overly complex or cumbersome to use [47,50]. In referencing the overall number of spheres on the home screen, many participants in this study found it overwhelming and complex to navigate and use, potentially particularly so, as one participant said, “on bad days.” Ease of use has been shown to be an important aspect of users' continued commitment to mHealth tools [99]. One feasible way of increasing the overall ease of use and user-friendliness could be to simplify the app by, for instance, allowing users to control the number of strengths spheres visible.

The literature on gameful mHealth design for well-being and mental health is still scarce [50,90]. In this study, ways of creating gameful designs without the use of proverbial game elements such as competitions or rewards have been presented. To help mature this field, future research should further explore approaches to engaging and gameful design in mHealth while also making sure these designs and features are thoroughly integrated into the tools in ways that are motivating, usable, and meaningful [49,90,100].

### Future Steps

Further development and efficacy testing of the actual MyStrengths app depends on funding. However, the following steps are recommended for future related research.

Based on this study, we wish to highlight 3 aspects related to the use and usability of MyStrengths that could be improved. First, an option to reduce the number of strengths spheres visible on the screen at any given time and keep their place on the screen constant (ie, disabling the spheres floating) could be beneficial. Along the same line, a form of notifications should be included. This could allow feedback to the users, such as summaries of strengths used in the past week, and also encourage app use, including reminding users to do strengths-exercises or the “three good things in life” activity. Third, the number of exercises included could also be increased, including a variation to suit as many users as possible (eg, including both physical and cognitive or mental tasks).

Despite the nonsignificant changes identified from the psychosocial outcome measures, several of these (eg, the General Self-Efficacy Scale, the Center for Epidemiologic Studies Depression Scale, and the General Strengths Use Scale) encouragingly yielded effect sizes in the small to medium range. Such effect sizes are not uncommon in psychosocial interventions (eg, Van Beugen et al [101]) and do not preclude potential clinical impact. For instance, in consideration of the possible reach of mHealth interventions, even small effect sizes can provide improvements to overall public health [102]. Although strengths use can impact people’s well-being and quality of life [14], the exact mechanisms still are somewhat unclear, and thus future research should further explore these aspects. Also, based on feedback from participants in this study, future research should explore the possibility that strengths-focused tools might be best suited for people new to life living with illness or unfamiliar with the strengths approach.

Given these findings, an expansion of the demographics or background questionnaire to include previous experiences with strengths-focused tools and interventions as well as including a posttrial questionnaire gauging use and impressions of the tools, design, and content could also be of benefit. The interviews in the current study mostly covered these aspects, but a larger number of participants included in future studies might allow inferences to be drawn concerning what types or groups of users (eg, based on age, gender, diagnosis, familiarity with strengths, or time with illness) strengths-focused mHealth tools could be best suited for.

This study has, using the MyStrengths app as a vehicle, demonstrated the feasibility of strengths assessment and exercises in unguided mHealth tools, and we hope the knowledge gained and shared from this work can be employed in future internal (ie, departmental) and external projects designing and testing mHealth solutions.

### Strengths and Limitations

This explorative feasibility study, combining interviews, outcome measures, and input from the system-use log, has some strengths and limitations. First, the gender balance of the participants in this study is heavily skewed, with 25 of 26 (96%) being women. Recruitment procedures strived to encourage more men to participate, unfortunately without success. Such imbalance of gender participation is not uncommon to mHealth research [103,104] and also mirrors the actual distribution of users of health care apps and digital services in Norway [105]. Second, as only users who had completed the 31-day trial were interviewed, we may lack feedback from users who potentially did not enjoy the app as much and therefore did not complete the trial.

On the other hand, it may be that these people already are thoroughly familiar with their strengths and could thus be considered outside the target group. Considering this, the participants interviewed in this study may even better represent the users. The heterogeneity of the participant group (eg, the variation in age, diagnosis, time with their illnesses) could also be considered a strength, as this allowed for a wide range of feedback and perspectives from the participants. Similarly, the fact that log data were only from participants completing the pre- and posttests (although the noncompleters shared the same basic background and demographical data) could be seen as a limitation, as the log data in this case may be skewed toward those “liking” the app.

Strengths-focused mHealth intervention is a new field with little existing research. Given this, what mechanisms are in play and how to measure these are still not entirely clear. None of the included outcome measures in the current study could yield solid information about how such a strengths-focused mHealth intervention could potentially impact users living with chronic illness. However, being an explorative pilot study with a relatively low number of participants (26) from diverse backgrounds, the primary goal of this pilot study was not to gauge any possible effects, and the result should thus not be unexpected. This also means that although some of the measures used showed medium effect sizes, no firm conclusion can be drawn as to whether the measures used are sensitive to the effects of using strengths-focused and positively oriented mHealth tools. Future research should explore underlying mechanisms or effects stemming from strengths interventions further in more extensive and more robust studies.

There is a need for both more holistic and robust research on eHealth and mHealth tools [61,106], and despite the described limitations, the mixed methods approach [60] in this study, using 3 data types (ie, the system-use log, the outcome measures, and participant interviews), provides a solid empirical backing allowing this study to provide important knowledge regarding the feasibility of strengths-focused mHealth apps in general and the MyStrengths app specifically.

### Conclusions

This study describes the explorative feasibility trial of the MyStrengths app, a mHealth tool that seeks to aid users in recognizing and employing their own strengths. MyStrengths is, to the best of our knowledge, the first mHealth tool of its kind. Through this feasibility trial, we have shown that all participants were able to identify a large number of their own strengths using the mHealth app. Although their preferences for exercises varied, most also found some they liked. The playful design elements (ie, the colored moving spheres and the die) were well received by parts of the participant group even though some also reported issues or limitations. There are also some indications that tools such as MyStrengths may be best suited for people relatively new to living with their illnesses. Based on this, future research should keep exploring the opportunities of both strengths-focused and gamefully designed mHealth apps.

Although impressions, reported usefulness, and feedback from use varied in this study, most participants reported being highly favorable to the strengths-focused approach to care and support. As such, mHealth tools such as MyStrengths may have the potential to support people living with chronic illness in a strengths-focused approach to self-management and mastery of their life. Further, as mHealth tools proverbially accompany users everywhere, creating strengths-focused mHealth tools may also increase the reach and availability of this form of support for those living with chronic illnesses.

## Appendix

Multimedia Appendix 1 Interview guide (translated from Norwegian to English).

## Abbreviations

mHealth: mobile health
SUS: System Usability Scale
