# Supplementary material for: Evaluating a Strengths-Based mHealth Tool (MyStrengths): Explorative Feasibility Trial
Source: JMIR Form Res. 2021 Nov 17;5(11):e30572. doi: 10.2196/30572 (PMC8663534; doi:10.2196/30572)
Supplement: Multimedia Appendix 1 [file formative_v5i11e30572_app1.docx]

# Interview guide MyStrenghts Feasibility trial. *(translated from Norwegian)*

Thank you for being part of evaluating the MyStrengths tool and for taking part in this interview. I will now go through a few questions concerning the app. We would appreciate it if you are as candid as possible, in particular concerning things that are negative or could be improved. The interview will take approximately 25. Minutes.

# General

- What did you think of the app?
  - What makes you think that?
  - Can you expand on that?
- What would you say is the goal of the app?
- What did you use it for?
- Did using the app give you anything?
- Have you used similar apps before?
  - Which?
  - In what situations?
- How did you find using the app?
  - And, how does this compare with others you have used?
- How much did you use the app?
  - Not much use:
    - What could perhaps have gotten you to use it more?
  - Much use:
    - What motivated you to use it?
- Where did you predominantly use it? (at home, on commutes, at the doctor?)
  - Why here?
- At what time during the day did you prefer using the app?
- Did you find using the app useful or worthwhile?
  - In what way?
  - Could it perhaps have been even more useful?
- If the app has not been used much at all
  - Why did you not use it?
  - Was it something about the app or something else?
  - Are there things or features that could have been in the app that would have made it more interesting, exciting, or relevant? That would have made you want to use it?

# Strengths

- How did you experience assessing your strengths?
  - Have you thought of your strengths before?
    - Is this something you are used to?
  - Have you done something similar to this assessment before?
    - What did you think of that?
    - What did you think of assessing your strengths in this manner compared to how you have done it before?
- How did you experience seeing the overview of your strengths in this manner?
- What did you think of the strengths that were included in the app?
  - Did you miss some?
  - Were any of them superfluous or out of place?
  - What do you think of the number of strengths?
- What do you think about the language used in the app?
- What do you think of the possibility of writing reflections connected to your strengths?
- What do you think of the possibility to create and do strengths-exercises?
- What did you think of the examples provided for the strengths-exercises?

# Design and Functionality

- What did you think of the daily-log/diary features?
  - Do you have any views on using smiley faces to rate your day?
  - What did you think of the “Three good things in life” exercise?
- Did you find using the app easy?
  - How did you find moving between the different functionalities such as Daily log, strengths, or strengths-exercises?
- Did you have any technical problems?
- Were there things you thought you could do in the app, but you were not able to?
- Did you want to explore the app when using it?
  - Or, did you for the most part just complete tasks and “do as you had been told”?
- Did you find anything particularly cool or engaging about it?
- Did you find anything particularly dull or lacking about it?
- What did you think about:
  - Using the spheres to visualize your strengths? (For instance, compared to a regular list)
  - The movement of the strengths-spheres
  - The different colors on the strengths-spheres
- Did you understand what the various colors represented?
- Do you plan to continue using the app?
  - Yes:
    - How do you think you will be using it going forward? (as before, or change something?)
    - When do you think you will be using it?
  - No:
    - Why not?
- Did you miss anything in the app?

# Ending

- Can you mention three things about MyStrenghts that you liked
- Can you mention three things about MyStrenghts that you disliked
- How did the app compare to the expectations you had for it?
- Did filling out the forms and surveys go OK?
- Do you have any other feedback, ideas, or questions for us?

Thank participant for the interview, and remind that they, of course still can contact us if they have any questions, etc.
